# Analysis of induced abortion-related complications in women admitted to the Kinshasa reference general hospital: a tertiary health facility, Democratic Republic of the Congo

**DOI:** 10.1186/s12978-018-0563-y

**Published:** 2018-07-06

**Authors:** Daniel Ishoso Katuashi, Antoinette Kitoto Tshefu, Yves Coppieters

**Affiliations:** 1 Community Health Department, Kinshasa School of Public Health, University of Kinshasa, PO Box11850, Kinshasa, Democratic Republic of Congo; 2 Research Centre “Policies and Health Systems - International Health”, School of Public Health, Université libre de Bruxelles (ULB), Brussel, Belgium

**Keywords:** Democratic Republic of the Congo, Complications of induced abortions, Analysis

## Abstract

**Background:**

Due to a lack of relevant data on induced abortions in the Democratic Republic of the Congo (DRC) as well as the persistence of maternal deaths in the country, this study aims to analyse the induced abortion-related complications in women who were admitted to the Kinshasa Reference General Hospital (KRGH).

**Methods:**

This is a cross-sectional study on 368 obstetric and gynecological patients who were admitted, as emergency cases, to the KRGH during 2014. This health facility was selected because it is a tertiary health facility with an obstetric and gynecological emergency unit most used in the city of Kinshasa. Patient data were collected from patient records and analyzed.

**Results:**

From the 368 patients admitted to receive obstetric and gynecological emergency care services in 2014 at the KRGH, 12.2% (95% CI: 9.1–16.1%) had complications due to induced abortion that was significantly diagnosed to adolescents (*p* <  0.001), single or separated or divorced women or widow(p <  0.001), and to patients with history of one or several induced abortions(p <  0.001). The median duration of hospitalization was ten days and this period of time was significantly longer for the patients who underwent surgery for pelvic peritonitis due to uterine perforation(*p* <  0.001) compared with the group of patients who underwent Caesarean section/hysterectomy.

The mortality rate related to them is 37.8% (95% CI: 23.8–53.5%) with an increase of risk of death in the presence of a post-abortive pelvic peritonitis-type complication, 56.3% of deaths occurred after two days of hospitalization.

**Conclusion:**

The complications of induced abortions are a major public health problem due to its frequency among patients admitted to the KRGH, as well as the poor medical management, and mortality percentage related to them. Therefore, there is a need to understand the reason for the poor medical management to fill in and provide an adequate intervention package.

## Plain English summary

Due to a lack of relevant data on induced abortions in the Democratic Republic of the Congo as well as the persistence of maternal deaths in the country, this study aims to analyse the induced abortion-related complications in women who were admitted to the Kinshasa Reference General Hospital (KRGH). This hospital was selected because it is a tertiary health facility with an obstetric and gynecological emergency unit most used in the city of Kinshasa.

Patient data were collected from patient records and analyzed.

From the 368 patients admitted to receive obstetric and gynecological emergency care services in 2014, 12.2% had complications due to induced abortion that was significantly diagnosed to adolescents, single or separated or divorced women or widow, and to patients with history of one or several induced abortions. The median duration of hospitalization was ten days, significantly longer for the patients who underwent surgery for pelvic peritonitis due to uterine perforation compared with the group of patients who underwent Caesarean section/hysterectomy.

The mortality rate related to them is 37.8% with an increase of risk of death in the presence of a post-abortive pelvic peritonitis-type complication, 56.3% of deaths occurred after two days of hospitalization.

In conclusion, the complications of induced abortions are a major public health problem due to its frequency, as well as the poor medical management, and mortality percentage related to them. Therefore, there is a need to understand the reason for the poor medical management to fill in and provide an adequate intervention package.

### Article summary

#### Strengths

A first survey to analyse the induced abortion-related complications in women who were admitted to the Kinshasa health facilities.

This survey is performed in a tertiary health facility with an obstetric and gynecological emergency unit most used in the city of Kinshasa.

#### Limitation

Firstly, the type of induced abortion complication did not often appear clearly in the physician’s diagnosis, and the clinical signs needed to be used to complete it.

Secondly, due to the lack of sufficient information in the records of patients who died for reasons other than induced abortions, it was very difficult for us to estimate the maternal mortality rate in this health facility.

## Background

About 25 million unsafe abortions are estimated to have taken place worldwide each year [1], the majority in developing countries, where about 7 million women are admitted to hospitals every year as a result of complications [2].

Complications from unsafe abortions account for about 7.9% (4.7–13.2%) of maternal deaths [3]. In absolute terms, approximately 193,000 maternal deaths occur each year worldwide, 192,000 in developing countries where sub-Saharan Africa has the largest share of 125,000. In addition to maternal deaths, high morbidity can be observed long-term such as premature births, psychic sequelae, infertility or subfertility, chronic pelvic pain, ectopic pregnancy, and spontaneous abortions [4–7].

In the Democratic Republic of the Congo, the prevalence of modern contraception is low at 8% [8], which probably explains the high rate of unwanted pregnancies at 147 per 1000 women of childbearing age and that of unsafe abortions at 57 per 1000 women of childbearing age [9]. In this country where: almost all induced abortions are backstreet abortions and unsafe because the laws are restrictive, the maternal mortality ratio remains very high at 846 deaths per 100,000 live births despite the improvement of many indicators of safe motherhood, such as the use of antenatal care services and assisted delivery supervised by trained personnel [8]; the occurrence of induced abortion complications, their admission to health facilities, and risk related deaths can be high. As these data are unavailable, this study proposes to analyze the cases of complication of induced abortion for patient who were admitted to the Kinshasa Reference General Hospital, which is a tertiary health facility with an obstetric and gynecological emergency unit most used in the city of Kinshasa according to the health information system data reported by Kinshasa health facilities in 2014 and available at the Provincial Health Division of Kinshasa. Specifically, the objectives of this study are: (i) to determine the proportion of induced abortion-related complications in women admitted to the obstetric and gynecological emergency unit of Kinshasa Reference General Hospital, as well as their demographic and socioeconomic characteristics; (ii) to measure the duration of hospitalization of the patients; and (iii) to determine the mortality rate due to induced abortion complications, their characteristics, as well as those that occur after two days of hospitalization.

## Methods

### Study design

In April 2015, a cross-sectional study was carried out at the Kinshasa Reference General Hospital. Data were collected retrospectively from 1st January 2014 to 31st December 2014.

### Study site

The Kinshasa Reference General Hospital is a tertiary health facility, with a capacity of 2000 beds, located in the administrative area of Gombe in Kinshasa. The bed occupancy rate in this hospital is 89.6, and 87.5% in the obstetric and gynecological department.

### Study population

The study population included in the study were women of childbearing age admitted to the obstetric and gynecological emergency unit of Kinshasa Reference General Hospital, from 1st January 2014 to 31st December 2014.

### Sampling

The Kinshasa Reference General Hospital is chosen because it is a tertiary health facility which has an obstetric and gynecological emergency unit most used in the city of Kinshasa according to the health information system data reported by Kinshasa health facilities in 2014 and available at the Provincial Health Division of Kinshasa.

All patients admitted to the obstetric and gynecological emergency unit of Kinshasa Reference General Hospital, from 1 January 2014 to 31 December 2014 were included.

The sample size was computed using the following formula: $\mathrm{n}\ge \frac{Z{\upalpha}^2.\mathrm{p}.\mathrm{q}}{d^2}$ where ‘p’ represents the proportion of induced abortion-related complications in women admitted to the obstetric and gynecological emergency unit of tertiary health facilities (we used the proportion of 27.6% found in an urban tertiary health facility in Nigeria [10] that gives the highest minimum size); q = 1-p; z the value of the standard normal distribution coefficient corresponding to a significance level of alpha of 0.05 (1.96) and; d the precision degree that we assumed to be at 5% too. The minimal size computed was 307 patients.

### Data collection, information sources, variables, and inclusion criteria

Data were collected by four nurses and one physician using a standardised investigation sheet. Information required to complete the investigation sheet was obtained from obstetric and gynecological emergencies and medical records.

The following variables were collected: demographic and social characteristics of patients (age, marital status, occupation); clinical characteristics of patients (temperature, parity, previous abortion, surgical history, clinical diagnosis, para-clinical explorations, treatment, length of hospital stay, outcome); and healthcare system variables (waiting time).

Regarding the variable age, the group of adolescents consisted of any subject aged 19 years or less, and this was in line with UNICEF, UNAIDS, and WHO definition.

Was included in the study, all patients admitted to the hospital for a gynecological and obstetrical problem (which implies a link with the pregnancy).

### Data processing and statistical analysis

Data were entered into an Epi Info software program version 3.5.4, exported to Microsoft Excel, and analyzed using Statistical Package for the Social Sciences (SPSS) version 20, IBM. Descriptive statistics were used to summarize the characteristics of the study population. Continuous variables were reported using mean with standard deviation for patient’s age, and median with interquartile range for length of hospital stay.

The Mann-Whitney test was used to compare the median length of hospitalization amongst the different groups of dichotomous variables examined. Categorical variables were reported as a frequency and percentage and groups were compared using the χ2 test. The forward stepwise logistic regression helped to identify independent predictors of induced abortion-related complications and deaths. All variables associated with induced abortion-related complications and deaths in the bivariate analysis were included in the final model. The odds ratio (OR) with a corresponding 95% confidence interval was reported to quantify the strength of association. Significance was set at *p*-value of less than 0.05.

### Ethical considerations

The National Committee of Ethics of the Kinshasa School of Public Health approved the present study. Authorization was also provided by health and politico-administrative authorities. Consent was obtained from the supervisor at Kinshasa Reference General Hospital prior to the collection of data. Anonymity was maintained during the collection of data and the survey forms of data collected were safeguarded.

## Results

### Frequency of cases of induced abortion-related complications admitted to Kinshasa reference general hospital

During 2014, three hundred and sixty-eight patients were admitted to the obstetric and gynecological emergency unit of Kinshasa Reference General Hospital, of which 12.2% (95% CI: 9.1–16.1%) had complications due to induced abortion (Table 1).

**Table 1:** Frequency of induced abortion-related complications cases

Patients admitted *n* = 368	n’ (%)	CI 95%
induced abortion-related complications cases		
Yes	45 (12.2)	9.1 to 16.1
No	323 (87.8)	84.0 to 90.9
Total	368 (100)	

With n = number of subjects in the sample; n’ = number of subjects in the sample subgroups

### Characteristics of induced abortion-related complications

The mean age of patient population was 29 years (± 7 years), and the majority of them was married or cohabiting, nulliparous, without a history of induced abortion. The adolescents had significantly a higher risk to experience an induced abortion complication than adults (Adjusted OR = 3.4; 95%CI: 1.1–10.1), the single or separated or divorced women or widows had significantly a higher risk than married women (Adjusted OR = 14.9; 95%CI: 6.0–36.8), and the patients with history of one or several induced abortions had significantly a higher risk than those who did not (Adjusted OR = 10.2; 95%CI: 3.7–28.2) (Table 2).

**Table 2:** Descriptive analysis

Variables	n’ (%)	Average (SD)
Patient’s residence (*n* = 359)		
Semi-rural quarters	20 (5.6)	
Eccentric quarters and of extension	66 (18.4)	
Quarters of planned cities	54 (15.0)	
Quarters of old cities	129 (35.9)	
Residential quarters	90 (25.1)	
Total	359 (100)	
Adolescence (years) (*n* = 362)		29 (7)
Yes	36 (9.9)	
No	326 (90.1)	
Total	362 (100)	
Marital status (*n* = 341)		
Married/cohabiting	271 (79.5)	
Single/separated/divorced/widowed	70 (20.5)	
Total	341 (100)	
Patient parity (*n* = 366)		
Nulliparous	141 (38.5)	
Primiparous	92 (25.1)	
Multiparous	133 (36.3)	
Total	366,100)	
Patient abortion background (n = 366)		
No abortion background	204 (55.7)	
One or several abortions	162 (44.3)	
Total	366 (100)	
Type of complications of induced abortion (*n* = 45)		
Post-abortion pelviperitonitis	24 (53.3)	
Others (infection, haemorrhage)	21 (46.7)	
Total	45 (100)	
Type of cases requiring surgery through abdomen (*n* = 347)		
Post-abortion pelviperitonitis	24 (6.9)	
Other diagnostics	323 (93.1)	
Total	347 (100)	

With: n = number of subjects in the sample; n’ = number of subjects in the sample subgroups; SD = Standard Deviation

Fifty three per cent of the cases of induced abortion complications were admitted for post-abortion pelviperitonitis.

In this study, the median duration of hospitalization was 10 days but was significantly longer for patients who underwent surgery for pelvic peritonitis due to uterine perforation (*p* <  0.001), compared with the group of patients who underwent Caesarean section/hysterectomy (Table 3).

**Table 3:** Demographic, social and clinical characteristics of the women admitted for induced abortion-related complications (bivariate and multivariate analysis)

	Induced abortion-related complications			
	Bivariate analysis		Multivariate analysis (LR)	
Independent variables	Crude OR (CI95%)	p	Adjusted OR (CI95%)	p
Adolescence (≤ to 19 yrs)		< 0.001		0.031
Yes (*n*’ = 36)	5.2 (2.4 to 11.2)		3.4 (1.1 to 10.1)	
No (*n*’ = 326)	1		1	
Patient’s residence		0.18		
Semi-rural quarters (*n*’ = 20)	2.4 (0.5 to 9.3)			
Residential quarters (*n*’ = 90)	2.3 (1.1 to 5.1)			
Eccentric quarters and of extension (n’ = 66)	1.2 (0.4 to 3.1)			
Quarters of planned cities (n’ = 54)	1.0 (0.3 to 1.9)			
Quarters of old cities (*n*’ = 129)	1			
Patient Marital Status		< 0.001		< 0.001
Single/separated/divorced/widowed (n’ = 70)	18.2 (8.4 to 41.8)		14.9 (6.0 to 36.8)	
Married/cohabiting (*n*’ = 271)	1		1	
Patient parity		0.4		
Nulliparous (*n*’ = 141)	1.6 (0.7 to 3.5)			
Primiparous (*n*’ = 92)	1.6 (0.7 to 3.8)			
Multi and grand multiparous (n’ = 133)	1			
Patient’s abortion background		< 0.001		< 0.001
One or several abortions (*n*’ = 162)	5.2 (2.5 to 10.8)		10.2 (3.7 to 28.2)	
No abortion background (*n*’ = 204)	1		1	

With: OR = Odds Ratio; P = p-value; LR = logistic regression

### Death characteristics

From the 45 patients admitted to the hospital due to induced abortion-related complications, 37.8% (95% CI: 23.8–53.5%) died, and 56.3% of deaths occurred after two days of hospitalization.

Furthermore, we found that there was an increased risk of dying for patients who had post-abortion pelviperitonitis (OR = 14.0; 95%CI: 4.9–39.9) compared with other gynecological and obstetric emergencies (Tables 4, 5 and 6).

**Table 4:** Duration of hospitalization for surviving women and analysis of variance according to the presence of induced abortion-related complication

Median duration of hospitalization (P25-P75) = 10 (8–13) days			
Variables	N	Median duration (P25-P75) of hospitalization (days)	p
Cases requiring a surgery through abdomen			< 0.001
Pelvic-peritonitis due to uterine perforation	13	21 (17–30)	
Other cases of surgery through abdomen	305	10 (8–13)

**Table 5:** Frequency of deaths related to complications of induced abortions and deaths occurred after two days of hospitalization

Patients admitted because of induced abortion-related complication n = 45	n’ (%)	CI 95%
deaths		
Yes	17 (37.8)	23.8 to 53.5
No	28 (62.2)	46.5 to 76.2
Total	45 (100)	
Patients admitted who died because of induced abortion-related complication *n* = 17	n’ (%)	CI 95%
Deaths after two days of hospitalization		
Yes	9 (56.3)	29.9 to 80.2
No	7 (43.7)	19.8 to 70.1
Total	16 (100)	
DDHA	1	

With: DDHA = data on the duration of hospitalization not available

**Table 6:** Characteristics of deaths related to complications of induced abortions

Variables	% Deaths	OR (CI95%)	P
Type of complications of induced abortion			0.13
Post-abortion pelviperitonitis (n’ = 24)	45.8	2.1 (0.6 to 7.7)	
Others (infection, haemorrhage) (n’ = 21)	28.6	1	
Type of Cases requiring a surgery through abdomen			< 0.001
Post-abortion pelviperitonitis (n’ = 24)	45.8	14.0 (4.9 to 39.9)	
Other cases of surgeries through abdomen (n’ = 323)	5.6	1	

## Discussion

In the study, 12.2% of patients admitted to the obstetric and gynecological emergency unit of Kinshasa Reference General Hospital had complications due to induced abortion. The findings of this study are similar to the results reported in Nigerian studies on urban areas [10, 11]. To the contrary, this diagnostic in rural tertiary health facility appeared to be relatively infrequent compared with those reported in urban areas. For example, the complications due to induced abortion diagnosed in a tertiary hospital located in the Niger Delta [12] and a large rural hospital located in the southwest of Nigeria [13] were 5.6 and 7.4%, respectively. Similar results were also reported in three sub-Saharan countries [14].

We have found a significant correlation between adolescence and the incidence of induced abortion-related complications. This finding is consistent with the results of many other studies carried out in comparable countries [10, 11, 13, 15–19].

A significant correlation with celibacy or separated or divorced women or widow was also observed in the present study, and these results corroborate those reported by many other authors [10, 11, 13, 16, 20–22]. These results differ from those of some authors who found a significant correlation with marriage or cohabitation: for example, in 2014 Dragoman’s secondary analysis of data from the World Health Organization (WHO), that may be correlated to the probable confusion that would be induced by cases of complications resulting from spontaneous abortions that had not been discriminated against in the database [15]; Igberase, at a tertiary hospital located in rural Nigeria [12], that may reflect possible rural realities; Shah at a University Hospital in Pakistan, and Erfani in Tehran, Iran [23, 24].

Furthermore, pelviperitonitis due to uterine perforation, constituted the most serious and urgent complication and accounted for 53% of all of the complications reported. ‘Sepsis’ was not the most common complication, as in other studies [23, 25].

In terms of the duration of hospitalization, the median length was 10 days for all surviving patients. However, a significantly extended hospitalization was observed for patients who had undergone a laparotomy for pelvic peritonitis, following uterine perforation; compared with patients who had undergone Caesarean section/hysterectomy. This result indicate that patients experiencing induced abortion-related complications had significantly longer hospital stays than patients with other comparable obstetric and gynecological complications, and that in the DRC this was representative of a greater cost of supportive care for the former group of patients.

In this study, the mortality rate due to induced abortion complications was 37.8%. Thus, induced abortion-related complications are a significant problem in the DRC, and this observation is consistent with the results of many other studies that were conducted in developing countries [11–13, 16, 23, 26–32]. It should also be noted that the lowest rates of mortality due to induced abortions are observed in countries where post-abortion care units have been established [33–39].

Regarding the characteristics of deaths, we find an increased risk of dying for patients with post-abortion pelviperitonitis compared with other gynecological and obstetric emergencies. These results suggest that the case management involving complications of abortions performed following laparotomy are inappropriately managed, since under the same operating conditions, the mortality rates were lower. Possibly, the deficiencies in supportive care consist of either inappropriate post-operative treatments or discrimination against these patients by medical staff members due to their inadequate perception of induced abortions. We also found that 56.3% of deaths occurred after two days of hospitalization. This latter finding supports our suspicion of insufficient supportive care for patients receiving aftercare for induced abortions, given that conventionally in the DRC, for patients who are admitted to hospital emergency units, deaths that occur after two days of hospitalization are attributable to the health facility, while those that occur earlier (within 1–2 days) can be attributable to a cause upstream of the health facility.

It should be noted that due to the lack of sufficient information in the records of patients who died for reasons other than induced abortions, it was very difficult for us to estimate the maternal mortality rate in this health facility.

## Conclusion

The results of this study demonstrate that induced abortion-related complications represent a major public health problem because of its frequency among the patients admitted to the Kinshasa Reference General Hospital, the poor medical management, and the deaths related to them. The hospital-based supportive care received by patients did not prevent nearly half of the deaths from occurring after two days of hospitalization, conventionally attributable to the health care system. Therefore, there is a need to understand the reason for the poor medical management to fill in and provide an adequate intervention package.

## Abbreviations

CI: Confidence interval
DRC: Democratic Republic of the Congo
F: Fisher’s exact test
n: Number of subjects in the sample
n’: Number of subjects in the sample subgroups
OR: Odds ratio
p: *p*-value
SD: Standard deviation
SPSS: Statistical package for the social sciences
WHO: World Health Organization
